# Plant Allometric Growth Enhanced by the Change in Soil Stoichiometric Characteristics With Depth in an Alpine Meadow Under Climate Warming

**DOI:** 10.3389/fpls.2022.860980

**Published:** 2022-05-09

**Authors:** Manhou Xu, Zitong Zhao, Huakun Zhou, Li Ma, Xiaojiao Liu

**Affiliations:** ^1^Institute of Geographical Science, Taiyuan Normal University, Jinzhong, China; ^2^Key Laboratory of Restoration Ecology of Cold Area in Qinghai Province, Northwest Institute of Plateau Biology, Chinese Academy of Sciences, Xining, China

**Keywords:** Qinghai-Tibetan Plateau, alpine meadow, plant allometric growth, soil stoichiometric characteristics, warming experiment

## Abstract

The effects of global warming have warmed the climate of the Qinghai-Tibetan Plateau (QTP) leading to changes in plant growth and soil nutrients in the alpine meadows. However, few studies have addressed the effects of warming on plant allometric growth and soil stoichiometry in these meadows on a long-term scale. Therefore, the effects of soil stoichiometry on plant allometric growth remain unclear under long-term warming in the alpine meadows. This study adopted infrared radiators to conduct an 8-year warming experiment in a permafrost region on the QTP starting in 2010, and surveyed growth indices of the plant community during the growing season. Soil organic carbon (C), total nitrogen (N), and total phosphorus (P) in an alpine meadow were measured. We initially learned that the aboveground part of the alpine meadow vegetation in the warming treatment changed from an isometric to an allometric growth pattern while the allometric growth pattern of the belowground part was further strengthened. Second, the contents of soil C, N, and P decreased at the 0–20 cm depth and increased at the 20–30 cm depth in warming. The ratios of soil C:N, C:P, and N:P showed increasing trends at different soil depths with artificial warming, and their amplitudes increased with soil depths. Warming promoted the migration of soil stoichiometric characteristics of C, N, and P to deep soil. Finally, the correlations of plant growth with soil stoichiometric characteristics were weakened by warming, demonstrating that the downward migration of soil stoichiometric characteristics to deep soil in warming had effects on the growth of vegetation in the alpine meadow. It concludes that the change in soil stoichiometric characteristics with soil depths promotes plant allometric growth in the alpine meadow under climate warming.

## Introduction

Temperature plays an important role in the process of plant growth and soil development (Saleska et al., 2002; Lee et al., 2020; Adamo et al., 2021). A rising temperature has irreversible effects on plant growth (Ma, 2015; Dixit et al., 2021). The pattern of plant growth mainly includes two types: isometric growth, which is expressed as a linear function, and allometric growth, which is expressed as a power function (Broadmeadow and Jackson, 2000). Plant allometric growth is a common phenomenon of disproportionate growth between two characteristics of an organism, which can reveal the inherent law of plant growth, but various species often have different growth patterns caused by the differences in genetic characteristics (Xu et al., 2021b). Therefore, the pattern of plant allometric growth mainly describes the non-linear quantitative relationship between the size of an individual or among the related attributes of an organism.

A large number of studies have been conducted on the pattern of plant allometric growth with the study objects focusing mostly on woody plants (Ma, 2015; Chen et al., 2016; Zhao et al., 2016; Yang et al., 2020). Through studying the relationships among plant growth indices, the allometric growth pattern can be revealed clearly among the structure, function, and physiological properties of woody plants (Ma, 2015). Furthermore, some studies have analyzed the allometric growth pattern of herbaceous plants, including analyzing the relationships among biomass, energy metabolism, nutrient content, and other growth indices (Zhao et al., 2020; Mahood et al., 2021). Although the allometric growth pattern was reflected completely among growth indices from woody plants to herbaceous plants, the plant growth patterns would change with different areas of distribution (Fei et al., 2016), types of trees (Wang X. et al., 2017), density (Lin et al., 2020), diameter class (Wang Y. et al., 2017), site environment (Lei et al., 2020), and competitive intensity (Li et al., 2011). That is, a transformation of the plant growth patterns would occur between the isometric and allometric growth patterns. For example, studies on different sandy land areas showed that the relationship between branch length and basal diameter of *Salix phellodendron* presented a pattern of allometric growth in mobile sandy land, and then adjusted to isometric growth in semi-fixed and fixed sandy land, and lastly changed to allometric growth again in inter-dune lowlands (Liu, 2017). Studies at different elevations have also shown that the relationship between stem length and stem biomass conformed to the pattern of allometric growth in high elevation areas, while the same species have a pattern of isometric growth in low elevation areas (He et al., 2021).

However, few reports were found on the response of the plant growth patterns to an increase in temperature in warming experiments, and fewer still have reported on the effects of warming on the allometric growth patterns. The response of plant growth to warming depended not only on the plant species and the hydrothermal environment but also on the duration of the warming experiment (Xu and Xue, 2013). Xu et al. (2015a,b, 2016) concluded that long-term warming not only affected the growth and development of plants but also changed the growth pattern caused by short-term warming. Therefore, it is important to study the effects of warming on plant growth patterns based on long-term warming experiments, especially the transformation between isometric and allometric growth in warming conditions.

Soil helps to stimulate plant growth, provides plants with their main source of required nutrients, and plays an important role in regulating and driving nutrient cycling in the ecosystems (Fu et al., 2010; Zhao F. Z. et al., 2015; Alatalo et al., 2017). Among these nutrients, soil carbon (C), nitrogen (N), and phosphorus (P) are the three most important factors affecting plant growth (Xu et al., 2015a). Previous studies using short-term warming experiments (<3 years) found that warming promoted the decomposition of organic matter by microorganisms, leading to a decrease in soil C content (Jiang et al., 2014). Other studies have found that warming limited the growth and reproduction of plants and belowground microorganisms, and an increase in plant litter led to an increase in the C and N content on the soil surface (Waldrop and Firestone, 2004), but the variation of the P content differed with different warming durations (Jiang et al., 2014). In the studies using long-term warming experiments (>5 years), warming caused soil organic matter and N content to tend to decrease over time, while the soil P content varied (Yang et al., 2015); however, other studies have found that the soil C content tended to increase under long-term warming (Gorissen et al., 2004). However, fewer studies have been conducted on the relationships between soil stoichiometric characteristics and plant growth patterns in warming experiments. Therefore, it is necessary to further study the changes in the characteristics of soil stoichiometric characteristics and their relationship to plant growth patterns in a long-term warming environment.

The Qinghai-Tibet Plateau (QTP) is known as the “third pole of the world” owing to its high elevation, unique climate type, and special sensitivity to climate warming (Feng et al., 1998; Julia et al., 2004). Alpine meadows on the QTP, where many species have adapted to the high elevations and long-term low temperature of the QTP, are extremely sensitive to global warming (Thomas et al., 2004; Xu and Xue, 2013). The climate of the plateau is growing warmer which is leading to changes in plant growth and soil nutrients (Ma et al., 2017). However, few studies have addressed the effects of warming on plant allometric growth and soil stoichiometry in alpine meadows at different time scales. Our previous study found that under short-term warming (1 year), an alpine meadow on the QTP exhibited allometric growth as a whole, but its aboveground part conformed to the characteristics of isometric growth, while belowground conformed to allometric growth (Xu and Xue, 2013). Additionally, we also learned that soil ammonium nitrogen and nitrate nitrogen had no obvious trends in different soil layers, but organic carbon, activated carbon, and total nitrogen all migrated to the deep soil and affected the proportion of root biomass in different soil layers on the alpine meadow (Xu et al., 2015a,^2016^). However, we still do not know how the plant allometric growth and soil stoichiometric characteristics of alpine meadows will change with long-term warming. It is also unclear whether the effects of soil stoichiometry on plant allometric growth at different warming time scales will change in the alpine meadow.

Therefore, based on an 8-year warming experiment at an alpine meadow of the QTP, we further explored the plant growth patterns and soil stoichiometric characteristics of the experimental meadow. The main contents were as follows: an analysis of (1) the changes in the plant growth patterns between isometric and allometric growth patterns, (2) the changes in the stoichiometric characteristics of soil C, N, and P, and (3) the correlations of the plant growth patterns with soil stoichiometric characteristics. By resolving these issues, we test a hypothesis that the allometric growth of an alpine meadow was influenced greatly by the migration of soil stoichiometric characteristics to deep soil during warming. These results can provide data support and theoretical guidance for the effective protection and rational use of alpine vegetation in high elevation areas, as well as the prevention of degradation or desertification in alpine grasslands.

## Materials and Methods

### Study Area

**FIGURE 1:**
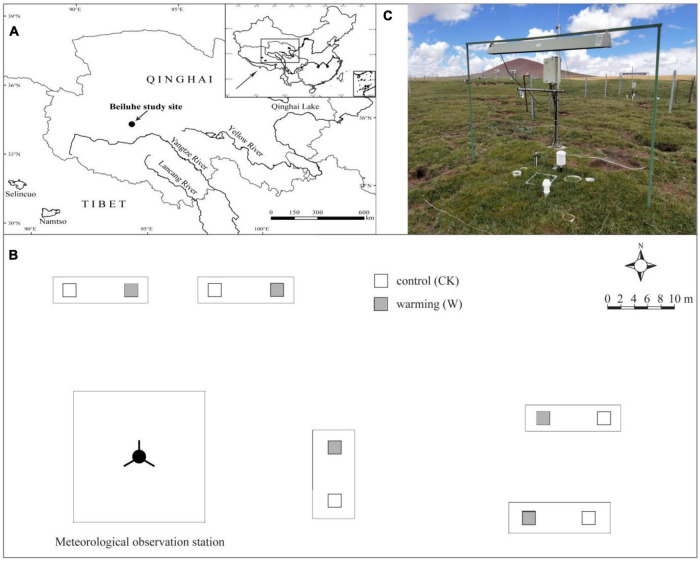
The research site on the Qinghai-Tibetan Plateau; **(A)** map of the site at the Beiluhe Station of Permafrost Engineering and Environment; **(B)** the experimental design with control and warming treatments; **(C)** photograph of a warmed plot with instrumentation in an alpine meadow.

The study area is located at the Beiluhe Station of Permafrost Engineering and Environment (herein, Beiluhe Station, Figure 1A), at 34°49′34″–34°49′37″N and 92°55′57″–92°56′06″E (Xu et al., 2015a,b, 2016). The elevation ranges from 4,620 to 4,640 m. From June 2010 to September 2018, the annual average temperature, precipitation, and relative humidity are −5.9°C, 267.6 mm, and 57%, respectively, while the annual potential evaporation is 1,316.9 mm (Qin et al., 2020; Wen et al., 2020). The rainfall is mainly concentrated from June to August while the wind direction is mainly from the northwest with an annual average wind speed of 4.1 m/s. The frost-free period ranges from April to September. The vegetation is that of a typical alpine meadow ecosystem, with *Kobresia pygmaea* as the dominant species as well as *Leontopodium nanum*, *Saussurea pulchra*, *Kobresia tibetica*, and *Oxytropis pusilla*. The plants of the Cyperaceae and Asteraceae account for a large part of the vegetation, whereas *Polygonum viviparum* (Polygonaceae) is also common (Ma X. et al., 2020). Soil development is weak and soils are classified as Mattic Cryic Cambisols (alpine meadow soil as Cambisols in the soil taxonomy of the Food and Agricultural Organization of the United Nations taxonomy) with a mattic epipedon at a depth of 0–10 cm and an organic-rich layer at 20–30 cm (Wang and Gu, 2021). The most common herbivore at the site is plateau pika (*Ochotona curzoniae*), which live in packs and burrow in soils (Liu et al., 2015).

### Experimental Design

The experimental plot was located in a typical alpine meadow about 300 m from the Beiluhe station. The vegetation was evenly distributed and the terrain was relatively flat. Infrared radiators (Figure 1B) were used in the experiment to truly simulate the enhanced downward infrared radiation the land would experience under future global warming. The energy radiated to the surface can change the surface temperature. A randomized block design was adopted in the experiment. Two treatments, control (CK) and warming (W), were established, with 5 replicates for each treatment and a total of 10 plots, with each plot area of 2 m × 2 m (Figure 1C). An infrared radiator was set up at a height of 1.5 m above the ground in the W plots, and only a lamp frame was set up for the CK plots to simulate the shading effects of the lamp frame on the vegetation in both the W and CK treatment.

An infrared surface temperature sensor (SI-111, Apogee Instruments, Inc., Logan, UT, United States) was set up at 1 m height from the ground to measure the ground surface temperature. A temperature and humidity probe (HMP45C, Campbell Scientific, Logan, UT, United States) was set up 20 cm above the ground to measure the air temperature and relative humidity at this height. A temperature probe (109SS-L, Campbell Scientific) was set in the soil to measure soil temperature at 0, 5, 15, 30, 60, and 100 cm depth. A data logger (CR1000, Apogee Instruments, Inc.) was used for data acquisition with a frequency of once every 10 min. A small ecological meteorological observation station was set up in the open area outside the W plots for conventional meteorological data observation. Both the W plots and the small eco-meteorological observation station were enclosed with barbed wire to prevent damage. The entire experimental plot was established in June 2010 (Xu et al., 2015a,b, 2016). The warming experiment started at 8:30 on 1 July 2010, and continued for 24 h a day; the amplitude of the temperature increase remained unchanged during the day and night. The warming experiment was conducted all year round for 8 years until 9:00 on 30 September 2018. During the 8 years, the air temperature and soil temperature increased by 0.19 and 1.23°C on average in the W plots in the alpine meadow, respectively (Qin et al., 2020; Wen et al., 2020).

### Investigation of Vegetation Characteristics

Community growth characteristics were investigated from late May to early September in 2011, 2012, 2013, 2016, 2017, and 2018 (Xu et al., 2015a,b, 2016; Qin et al., 2020; Wen et al., 2020). In the 8 years, the data from 2014 and 2015 were missed as there were no field works in this period although the work continued again in 2016. Sample frames of 27 cm × 27 cm (side width of 2 cm and internal size of 25 cm × 25 cm) were used to measure plant growth indices. The frame was divided into 100 grids of 2.5 cm × 2.5 cm based on the size of the hard wire mesh. The surveyed plant indices included density, height, coverage, frequency, aboveground biomass, and belowground biomass (Table 1). First, we determined the characteristics of each species in the frame, and the species diversity indices were then calculated using the characteristic values. We then selected some temporary plots around the experimental CK and W plots. The plant height and coverage in the temporary plots were consistent with the experimental plots. The aboveground biomass of the temporary plots was harvested, transported to the laboratory, placed into an oven, dried for 24 h to a constant weight at 75°C, and then weighed. Finally, a stepwise regression equation was established using the height, coverage, and aboveground biomass of the plants in the temporary plots, and the aboveground biomass in the experimental plots was indirectly obtained.

**TABLE 1:** Plant and soil characteristics under control (CK) and warming (W) treatments along different years in an alpine meadow.

Plant	Density (plant/m^2^)		Height (m)		Frequency (%)		Coverage (%)		Aboveground biomass (g/m^2^)		Belowground biomass (g/m^2^)	
	CK	W	CK	W	CK	W	CK	W	CK	W	CK	W
2011	784.742 ± 68.897	448.908 ± 32.574	0.022 ± 0.005	0.024 ± 0.007	18.668 ± 1.491	17.450 ± 2.663	7.146 ± 0.407	5.122 ± 0.418	176.862 ± 24.309	211.448 ± 22.259	8848.850 ± 985.080	9797.962 ± 333.092
2012	801.738 ± 128.082	840.046 ± 175.858	0.024 ± 0.002	0.038 ± 0.003	24.598 ± 3.228	28.090 ± 4.751	7.450 ± 0.859	7.808 ± 1.213	201.886 ± 10.631	216.414 ± 13.840	3889.244 ± 571.276	4543.818 ± 585.846
2013	813.777 ± 64.505	604.000 ± 97.780	0.020 ± 0.001	0.020 ± 0.005	31.967 ± 4.153	21.000 ± 3.442	7.550 ± 0.473	6.000 ± 0.514	195.747 ± 5.024	182.630 ± 10.188	3383.337 ± 458.519	3465.747 ± 150.458
2016	740.236 ± 77.434	880.654 ± 329.207	0.044 ± 0.004	0.046 ± 0.005	26.416 ± 3.141	21.144 ± 2.256	12.352 ± 2.199	11.414 ± 1.744	282.606 ± 49.366	282.026 ± 29.319	1057.064 ± 76.773	986.870 ± 39.513
2017	1413.228 ± 686.547	1383.748 ± 509.580	0.038 ± 0.004	0.050 ± 0.006	22.400 ± 3.056	20.076 ± 3.220	10.514 ± 2.303	10.246 ± 2.407	255.522 ± 57.532	301.130 ± 77.450	1061.794 ± 62.641	1113.480 ± 241.531
2018	415.756 ± 86.409	559.352 ± 148.043	0.040 ± 0.006	0.058 ± 0.003	22.366 ± 2.321	26.638 ± 3.199	9.428 ± 1.127	9.558 ± 1.269	230.454 ± 45.737	268.724 ± 27.043	11675.498 ± 1132.369	11628.962 ± 962.081

Soil	Carbon content at the 0–10 cm depth (g/kg)		Carbon content at the 10–20 cm depth (g/kg)		Carbon content at the 20–30 cm depth (g/kg)		Nitrogen content at the 0–10 cm depth (g/kg)		Nitrogen content at the 10–20 cm depth (g/kg)		Nitrogen content at the 20–30 cm depth (g/kg)		Phosphorus content at the 0–10 cm depth (g/kg)		Phosphorus content at the 10–20 cm depth (g/kg)		Phosphorus content at the 20–30 cm depth (g/kg)	
	CK	W	CK	W	CK	W	CK	W	CK	W	CK	W	CK	W	CK	W	CK	W
2011	7.607 ± 2.159	5.119 ± 1.023	6.176 ± 1.784	4.138 ± 0.718	4.811 ± 1.699	4.340 ± 0.645	0.729 ± 0.147	0.564 ± 0.323	0.596 ± 0.108	0.465 ± 0.043	0.467 ± 0.111	0.465 ± 0.051	0.395 ± 0.022	0.344 ± 0.018	0.345 ± 0.016	0.345 ± 0.013	0.365 ± 0.032	0.375 ± 0.016
2012	6.964 ± 1.296	6.330 ± 0.952	5.428 ± 0.708	5.552 ± 0.582	4.508 ± 0.726	5.652 ± 0.742	0.638 ± 0.107	0.584 ± 0.091	0.488 ± 0.056	0.508 ± 0.054	0.400 ± 0.068	0.496 ± 0.065	0.370 ± 0.027	0.345 ± 0.020	0.349 ± 0.014	0.347 ± 0.012	0.344 ± 0.018	0.362 ± 0.014
2013	8.587 ± 0.687	7.503 ± 0.913	6.963 ± 1.313	5.787 ± 0.503	5.190 ± 0.938	5.647 ± 0.334	0.781 ± 0.071	0.695 ± 0.083	0.655 ± 0.117	0.564 ± 0.047	0.496 ± 0.082	0.559 ± 0.041	0.406 ± 0.019	0.368 ± 0.018	0.393 ± 0.029	0.361 ± 0.010	0.371 ± 0.021	0.379 ± 0.009
2016	15.080 ± 2.813	14.425 ± 2.427	11.324 ± 1.841	11.751 ± 1.388	9.656 ± 1.650	10.668 ± 1.035	0.983 ± 0.159	0.947 ± 0.133	0.754 ± 0.110	0.794 ± 0.093	0.715 ± 0.108	0.750 ± 0.056	0.436 ± 0.020	0.405 ± 0.014	0.397 ± 0.011	0.389 ± 0.012	0.390 ± 0.029	0.378 ± 0.017
2017	8.815 ± 1.539	10.036 ± 1.942	7.983 ± 1.432	7.957 ± 1.359	6.250 ± 1.074	6.653 ± 1.118	0.977 ± 0.138	1.139 ± 0.185	0.862 ± 0.135	0.889 ± 0.126	0.742 ± 0.092	0.816 ± 0.087	0.480 ± 0.027	0.474 ± 0.020	0.451 ± 0.030	0.433 ± 0.018	0.438 ± 0.037	0.455 ± 0.030
2018	14.482 ± 2.354	13.750 ± 2.158	10.721 ± 2.263	11.798 ± 1.976	10.496 ± 2.361	9.891 ± 1.731	1.092 ± 0.153	1.053 ± 0.141	0.789 ± 0.106	0.826 ± 0.105	0.802 ± 0.136	0.709 ± 0.096	0.462 ± 0.017	0.429 ± 0.019	0.415 ± 0.011	0.416 ± 0.025	0.426 ± 0.033	0.407 ± 0.017

*The data are shown by mean ± standard error.*

The plant belowground biomass was sampled using a soil corer with an internal diameter of 7 cm in late July each year. One core was extracted every 10 cm between 0 and 30 cm depth in the center of each experimental plot, providing three soil layers (0–10, 10–20, and 20–30 cm). After the root and soil materials were extracted, they were immediately placed in a cooler and transported to the laboratory. In the laboratory, the soil samples were air-dried and crumbled by hand to pass through a 2-mm diameter sieve to remove large particles from the finer soil. Subsequently, the roots were separated from the soil samples and filtered with a 0.25 mm sieve to retrieve fine roots. Live roots were distinguished from dead roots by their colors, consistency, and the presence or absence of attached fine roots (Yang et al., 2009, 2010; Xu et al., 2015a,^2016^). The live roots were put into an oven to dry for 48 h until a constant weight at 75°C, and then plant belowground biomass was recorded.

The total biomass is the sum of the aboveground and belowground biomass. The root to shoot ratio (R:S) is the ratio of belowground to aboveground biomass.

### Measurement of Soil Nutrients

After sieving out the plant roots, the remaining soil was used to measure the C, N, and P content at depths of 0–10, 10–20, and 20–30 cm (Table 1). Among them, soil organic carbon (C) was determined by the potassium permanganate oxidation method (external heating method), the total nitrogen (N) was determined with the Kjeldahl method, and the total phosphorus (P) was determined by the vanadium molybdate blue colorimetric method (Xu et al., 2015a; Zhang et al., 2017).

### Data Analysis

Because the warming experiment was designed for the long term, 100 temporary plots with similar plant height and coverage were established near the experimental plots to estimate the aboveground biomass on the experimental plots during the growing season from May 2010 to September 2013 (Xu et al., 2015b,^2016^; Wen et al., 2020). The plant aboveground biomass data in the warmed plots were calculated at the community level by establishing a stepwise regression equation (Xu et al., 2015b,^2016^; Wen et al., 2020) using plant height, coverage, and aboveground biomass from the temporary plots in the SPSS 23.0 software (IBM Corp., Armonk, United States):


$$\begin{array}{c} \text{AGB}=308.26c+22.764h-121.801\\ \;\left(R^{2}=0.737,p<0.001,N=100\right), \end{array}$$

where AGB is the plant aboveground biomass (g/m^2^), *c* is the plant coverage (fractional representation), and *h* is the plant height (cm); *R*^2^ is the determination coefficient, *P* is the test level of significance, and *N* is the total number of temporary plots.

Raw data (Table 1) were transformed or calculated to different indices in Excel 2020, including eight indices on plant growth (density, height, coverage, frequency, aboveground biomass, belowground biomass, total biomass, and the plant R:S ratio), six indices on the soil stoichiometric characteristics at three depths (contents of C, N, and P elements as well as their ratios of C:N, C:P, and N:P), six indices on warming time (years of 2011, 2012, 2013, 2016, 2017, and 2018), and two indices on experimental treatment (CK and W).

First, for the plant growth indices, regression analysis software in the SPSS 23.0 software was used to explore the relationship between plant biomass indices (aboveground biomass, belowground biomass, and total biomass) and other plant growth indices (density, height, frequency, and coverage) in CK and W treatments, and the optimal equation was selected from the following conventional functions: exponential, linear, logarithmic, quadratic polynomial, and power functions (Xu et al., 2021a,b). The selection criteria had two conditions: (1) the determination coefficient of *R*^2^ value was the largest and (2) the significance test of the *p-*value was the smallest and less than 0.05 (Xu et al., 2021a,b). The optimal fitting plots were drawn with Origin 9.1 software (OriginLab Corp., Hampton, MA, United States). By exploring these relationships between plant growth indices, we could determine the type of plant growth patterns (isometric or allometric growth) in different treatments, and then determine the transformation between them in the W treatments.

Second, for the soil stoichiometric characteristics of C, N, and P elements, a two-way analysis of variance in the SPSS 23.0 software and the coefficient of variation (CV) were adopted to probe the trends of soil C, N, and P contents together with their ratios of C:N, C:P, and N:P at three different soil depths. The classes of CV were divided into three types: (1) small (from 0 to 15%), medium (15–35%), and large (>36%) variation (Qin et al., 2020). The bar graphs of the soil stoichiometric characteristics at different soil depths were also drawn with the Origin 9.1 software.

Finally, Canoco for Windows 4.5 software (Biometis, Wageningen, Netherlands) was used to conduct detrended correspondence analysis on data of plant growth indices to find any correlations between plant growth indices and soil stoichiometric characteristics; then, a linear model was conformed to process direct gradient analysis because the maximum gradient length of the ordination axes was less than 3, so redundancy analysis was chosen to conduct constraint sequencing analysis.

## Results

### Effects of Warming on Plant Growth

**FIGURE 2:**
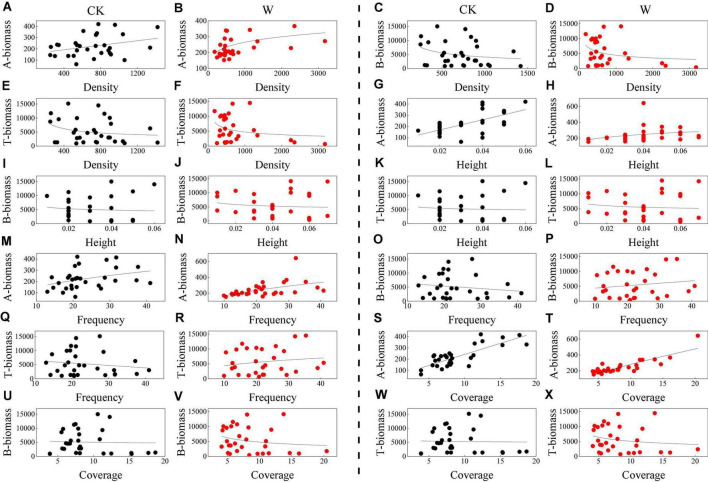
The optimal correlations of plant biomass with **(A–F)** plant density, **(G–L)** height, **(M–R)** frequency, and **(S–X)** coverage under control (CK), and warming (W) treatments in an alpine meadow. A-biomass, B-biomass, and T-biomass indicate the aboveground, belowground, and total biomass, respectively. For each index (plant density, height, frequency, and coverage), CK and W graphs are shown in sequence for A-biomass, B-biomass, and T-biomass. For example, **(A,B)** show A-biomass for CK and W treatments followed by **(C,D)** for B-biomass for CK and W treatments.

By fitting the optimal equations among plant growth indices in all treatments, the power function, with the power exponents varying from −1.097 to 0.570, was found to be the best one to explain the growth pattern of the alpine meadow vegetation (Figure 2 and Table 2).

**TABLE 2:** The optimal functional equation of plant biomass with plant density, height, frequency, and coverage under control (CK) and warming (W) treatments in an alpine meadow.

Index	Treatment	A-biomass (g/m^2^)	B-biomass (g/m^2^)	T-biomass (g/m^2^)
Density (plant/m^2^)	CK W	*y* = 0.0491*x* + 184.08 (*R*^2^ = 0.1447, *P* < 0.001) *y* = 70.917*x*^0.1856^ (*R*^2^ = 0.1657, *P* < 0.001)	*y* = 87680*x*^–0.499^ (*R*^2^ = 0.0821, *P* < 0.001) *y* = 5810.1e^–0.001^*^x^* (*R*^2^ = 0.1835, *P* < 0.001)	*y* = −2052ln(*x*) + 18,679 (*R* ^2^ = 0.0719, *P* < 0.001) *y* = 5962.8e^–0.001^*^x^* (*R*^2^ = 0.1579, *P* < 0.001)
Height (m)	CK W	*y* = 4530.3*x* + 78.777 (*R*^2^ = 0.4054, *P* < 0.001) *y* = 494.4*x*^0.2273^ (*R*^2^ = 0.1667, *P* < 0.001)	*y* = 428.65*x*^–0.587^ (*R*^2^ = 0.0699, *P* < 0.001) *y* = 931.5*x*^–0.401^ (*R*^2^ = 0.0435, *P* < 0.001)	*y* = 727.11*x*^–0.465^ (*R*^2^ = 0.0526, *P* < 0.001) *y* = 1297.1*x*^–0.335^ (*R*^2^ = 0.0378, *P* < 0.001)
Frequency (%)	CK W	*y* = 34.671*x*^0.5699^ (*R*^2^ = 0.1428, *P* < 0.001) *y* = 57.779*x*^0.4601^ (*R*^2^ = 0.3338, *P* < 0.001)	*y* = −89.73*x* + 7209.5 (*R*^2^ = 0.0231, *P* < 0.001) *y* = 81.032*x* + 3592.5 (*R*^2^ = 0.0244, *P* < 0.001)	*y* = −85.355*x* + 7330.4 (*R*^2^ = 0.021, *P* < 0.001) *y* = 2550.5e^0.0196^*^x^* (*R*^2^ = 0.0302, *P* < 0.001)
Coverage (%)	CK W	*y* = 19.676*x* + 45.912 (*R*^2^ = 0.6359, *P* < 0.001) *y* = 133.5e^0.0662^*^x^* (*R*^2^ = 0.7613, *P* < 0.001)	*y* = 6384.6e^–0.07^*^x^* (*R*^2^ = 0.075, *P* < 0.001) *y* = 16089*x*^–0.747^ (*R*^2^ = 0.0948, *P* < 0.001)	*y* = 5917.4e^–0.05^*^x^* (*R*^2^ = 0.0468, *P* < 0.001) *y* = 13300*x*^–0.599^ (*R*^2^ = 0.076, *P* < 0.001)

*Since the p-value of all fitting equations is less than 0.001, the optimal equation was selected according to the maximum R^2^-value. A-biomass, aboveground biomass; B-biomass, belowground biomass; T-biomass, total biomass.*

Under the CK treatment, the aboveground biomass showed a power function relationship with frequency (power exponent of 0.5699) (Figure 2M) and showed linear function relationships with density, height, and coverage (Figures 2A,G,S). The relation of belowground biomass had an exponential function and a linear function with the coverage and frequency (Figures 2U,O), respectively, whereas power functions with both density and height (Figures 2C,I), with the corresponding power exponent being −0.499 and −0.587 (Table 2). The total biomass was negatively correlated with density, height, frequency, and coverage (Figures 2E,K,Q,W). The relationship between the total biomass and height was a power function with a power exponent of −0.465 (Table 2), so the alpine meadow was in the pattern of allometric growth. In conclusion, in the control treatment, the aboveground part of the alpine meadow vegetation showed an isometric growth pattern, while the belowground part showed an allometric growth pattern, and the whole of the vegetation showed an allometric growth pattern.

Under the warming treatment, the power exponents of the aboveground biomass were all positive with density, height, and frequency (Figures 2B,H,N). However, the power exponents of density, height, and frequency were all small, ranging from 0.18 to 0.46 (Table 2). The correlations of belowground biomass showed power functions with height and coverage (Figures 2J,V), with power exponents of −0.401 and −0.747, respectively (Table 2). The correlation presented a linear model between belowground biomass and frequency (Figure 2P), which was consistent with the pattern of isometric growth. The relationships of total biomass were exponential functions with density and frequency (Figures 2F,R), while were power functions with height and coverage (Figures 2L,X) with the power exponents being −0.335 and −0.599 (Table 2). In general, under the W treatment, both aboveground and belowground parts of the alpine meadow vegetation conformed to the pattern of allometric growth.

### Effects of Warming on Soil Stoichiometric Characteristics

**FIGURE 3:**
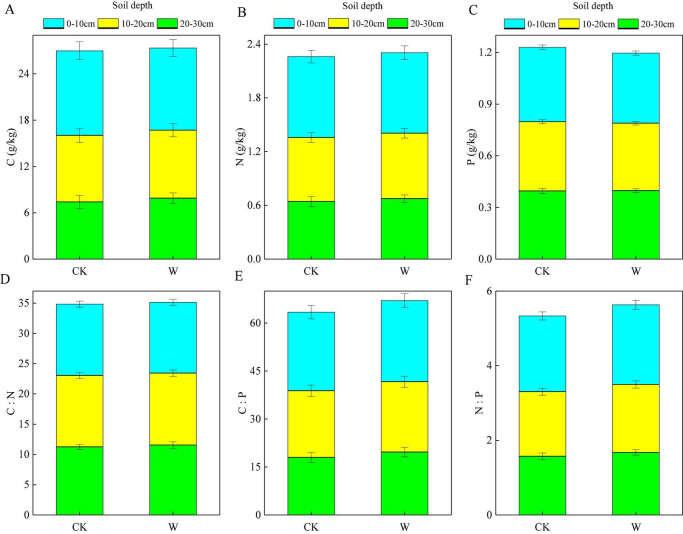
Effects of warming on the soil stoichiometric characteristics with **(A)** organic carbon (C), **(B)** total nitrogen (N), and **(C)** total phosphorus (P) at three soil depths in an alpine meadow. **(D)** C:N, **(E)** C:P, and **(F)** N:P denote the content ratios of C to N, C to P, and N to P, respectively. The three pieces of each column from top to bottom represent the values of the soil stoichiometric characteristics at soil depths of 0–10, 10–20, and 20–30 cm, respectively.

Among all soil elements, the P content was the most stable (CV = 15.23%), while the C (CV = 52.12%) and N (CV = 40.56%) contents changed dramatically (Table 3). The C, N, and P contents varied insignificantly in response to warming (*p* > 0.05) (Table 3). Under the W treatment, the C and N contents increased by 0.18 and 1.22% (Figures 3A,B), respectively, while the P content decreased by 2.70% (Figure 3C). For different soil depths in warming, the C, N, and P contents decreased by 5.94, 3.36, and 6.39% at the 0–10 cm depth (Figures 3A–C), respectively. At the 10–20 cm depth, the C and P contents decreased by 0.56 and 2.81% (Figures 3A,C), respectively, while the N content increased by 0.17% (Figure 3B). At the 20–30 cm depth, the C, N, and P contents increased by 10.84, 9.97, and 1.43%, respectively (Figures 3A–C). Therefore, the contents of C, N, and P decreased at the 0–20 cm depth and increased at the 20–30 cm depth in warming, indicating that warming forced the C, N, and P elements to have tendencies to transfer to deep soil in the alpine meadow.

**TABLE 3:** Effects of warming and depth on the soil stoichiometric characteristics with organic carbon (C), total nitrogen (N), and total phosphorus (P) in an alpine meadow.

Method	Treatment	C	N	P	C:N	C:P	N:P
CV (%)	Warming	52.12	40.56	15.23	20.94	42.57	28.23
ANOVA	Warming	0.877	0.774	0.283	0.803	0.430	0.227
	Depth	0.004	<0.001	0.112	0.640	0.005	<0.001
	Warming × Depth	0.914	0.957	0.569	0.917	0.971	0.996

*C:N, C:P, and N:P express the content ratios of C to N, C to P, and N to P, respectively. CV, coefficient of variation; ANOVA, analysis of variance.*

Among all the soil element ratios, the C:N ratio was the most stable (CV = 20.94%), while the C:P ratio changed dramatically (CV = 42.57) (Table 3). Under the W treatment, the C:N ratio increased by 0.13% (Figure 3D), the C:P ratio increased by 4.06% (Figure 3E), and the N:P ratio increased by 4.65% (Figure 3F), but these changes were all not significant (*p* > 0.05; Table 3). For different soil depths in the W treatment, the C:N decreased by 1.10% at the 0–10 cm depth and increased by 0.89 and 2.32% at the depths of 10–20 and 20–30 cm, respectively (Figure 3D). The C:P ratio increased by 1.06, 3.28, and 11.49% at the depths of 0–10, 10–20, and 20–30 cm, respectively (Figure 3E). The N:P ratio increased by 3.24, 3.97, and 9.72% at the depths of 0–10, 10–20, and 20–30 cm, respectively (Figure 3F). Therefore, these three ratios showed increasing trends at different soil depths in the W treatment, and the amplitudes increased with depths, indicating that warming promoted the C:N, C:P, and N:P ratios to migrate to deep soil in the alpine meadow as well.

### Correlation Between Plant Growth and Soil Stoichiometric Characteristics in Warming

Significant correlations were all positive between the plant growth and soil stoichiometric characteristics, indicating that soil stoichiometric characteristics facilitated the plant growth patterns in the alpine meadow; however, the correlations of plant growth from the CK treatment to the W treatment were diverse with the soil stoichiometric characteristics following by increasing temperatures (Table 4). First, warming enhanced the correlations between plant density and soil stoichiometric characteristics; second, warming weakened the correlations of plant height, coverage, and aboveground biomass with soil stoichiometric characteristics; and third, warming had no obvious influences on the correlations of plant frequency and belowground biomass with soil stoichiometric characteristics.

**TABLE 4:** Effects of warming on the correlations of plant growth with the soil stoichiometric characteristics in an alpine meadow.

Index	Treatment	De	He	Fr	Co	Ab	Bb	Tb	Rs
C1	CK	0.047	0.525*	0.363	0.685**	0.559**	0.305	0.318	0.152
	W	0.305	0.390	0.362	0.586**	0.453*	0.233	0.244	0.132
C2	CK	0.196	0.572**	0.359	0.680**	0.616**	0.267	0.281	0.112
	W	0.332	0.450*	0.418*	0.581**	0.456*	0.307	0.318	0.157
C3	CK	0.055	0.548**	0.262	0.580**	0.470*	0.434*	0.445*	0.340
	W	0.293	0.401	0.393	0.511*	0.374	0.279	0.288	0.134
N1	CK	0.242	0.486*	0.421*	0.714**	0.554**	0.288	0.302	0.170
	W	0.425*	0.421*	0.355	0.583**	0.557**	0.136	0.150	0.015
N2	CK	0.475*	0.474*	0.444*	0.728**	0.636**	0.110	0.124	<0.001
	W	0.580**	0.465*	0.402	0.635**	0.612**	0.087	0.101	–0.068
N3	CK	0.245	0.572**	0.273	0.639**	0.534**	0.304	0.316	0.227
	W	0.495*	0.440*	0.287	0.494*	0.441*	0.010	0.021	–0.111
P1	CK	0.402	0.384	0.338	0.597**	0.418*	0.159	0.169	0.088
	W	0.410	0.436*	0.215	0.513*	0.494*	0.043	0.055	–0.099
P2	CK	0.404	0.317	0.364	0.572**	0.478*	0.024	0.035	–0.036
	W	0.346	0.544**	0.220	0.362	0.343	0.148	0.156	0.105
P3	CK	0.159	0.425*	0.228	0.558**	0.563**	0.210	0.224	0.055
	W	0.465*	0.415*	0.249	0.341	0.320	0.028	0.036	–0.057
CN1	CK	–0.225	0.358	0.213	0.398	0.323	0.197	0.205	0.090
	W	–0.087	0.197	0.110	0.221	0.086	0.212	0.214	0.197
CN2	CK	–0.190	0.470*	0.164	0.363	0.324	0.288	0.296	0.180
	W	–0.168	0.277	0.182	0.221	0.090	0.397	0.400	0.332
CN3	CK	–0.248	0.229	0.214	0.278	0.191	0.386	0.391	0.315
	W	–0.120	0.215	0.282	0.241	0.129	0.437*	0.440*	0.348
CP1	CK	–0.041	0.505*	0.373	0.655**	0.523*	0.293	0.305	0.149
	W	0.229	0.341	0.354	0.553**	0.393	0.239	0.249	0.160
CP2	CK	0.109	0.576**	0.342	0.639**	0.557**	0.294	0.307	0.149
	W	0.299	0.364	0.421*	0.608**	0.466*	0.282	0.293	0.101
CP3	CK	0.031	0.539**	0.308	0.561**	0.358	0.425*	0.434*	0.401
	W	0.151	0.320	0.333	0.471*	0.325	0.258	0.266	0.123
NP1	CK	0.143	0.484*	0.464*	0.706**	0.532**	0.314	0.326	0.197
	W	0.402	0.394	0.417*	0.613**	0.553**	0.171	0.185	0.060
NP2	CK	0.422*	0.520*	0.460*	0.733**	0.614**	0.159	0.173	0.046
	W	0.621**	0.374	0.456*	0.717**	0.676**	0.049	0.065	–0.155
NP3	CK	0.292	0.615**	0.314	0.633**	0.418*	0.294	0.304	0.300
	W	0.394	0.370	0.253	0.529**	0.445*	–0.033	–0.023	–0.160

De, plant density; He, plant height; Fr, plant frequency; Co, plant coverage; Ab, plant aboveground biomass; Bb, plant belowground biomass; Tb, plant total biomass; Rs, plant root and shoot ratio. In the following, 1, 2, and 3 represent soil depths of 0–10, 10–20, and 20–30 cm, respectively. C1, C2, and C3 are soil carbon content; N1, N2, and N3 are soil nitrogen content; P1, P2, and P3 are soil phosphorus content; CN1, CN2, and CN3 are the soil carbon and nitrogen ratios; CP1, CP2, and CP3 are the soil carbon and phosphorus ratios; NP1, NP2, and NP3 are the soil nitrogen and phosphorus ratios. CK and W are the control and warming treatments, respectively. * and ** Show significant differences at the 0.05 and 0.01 levels, respectively. The data are correlation coefficients.

**FIGURE 4:**
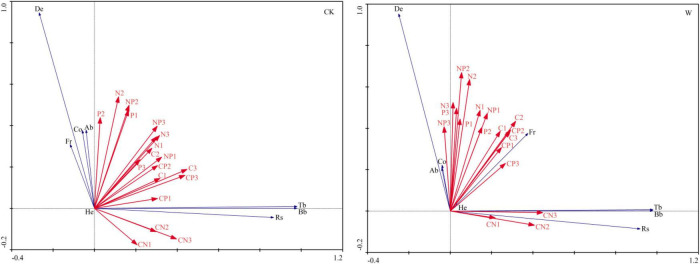
Redundancy analyses of the relationships between plant growth indices and soil stoichiometric characteristics under control (CK) and warming (W) treatments in an alpine meadow. De, plant density; He, plant height; Fr, plant frequency; Co, plant coverage; Ab, plant aboveground biomass; Bb, plant belowground biomass; Tb, plant total biomass; Rs, plant root and shoot ratio. In the following, 1, 2, and 3 represent soil depths of 0–10, 10–20, and 20–30 cm, respectively. C1, C2, and C3 are soil carbon content; N1, N2, and N3 are soil nitrogen content; P1, P2, and P3 are soil phosphorus content; CN1, CN2, and CN3 are the soil carbon and nitrogen ratios; CP1, CP2, and CP3 are the soil carbon and phosphorus ratios; NP1, NP2, and NP3 are the soil nitrogen and phosphorus ratios.

The redundancy analysis also revealed that the correlations between the plant growth and soil stoichiometric characteristics were weakened by warming, demonstrating the downward migration of the soil stoichiometric characteristics in warming creating influences on the growth of aboveground vegetation in the alpine meadow (Figure 4). For different soil depths, warming mainly enhanced correlations of plant growth with soil stoichiometric characteristics at the 10–30 cm depth, which further proved that the migration of soil stoichiometric characteristics to deep soil, caused by warming, had influenced the growth of vegetation in the alpine meadow (Table 4).

## Discussion

### Responses of the Plant Growth Patterns to Warming

In this study, the power function was more suitable to describe the plant growth patterns of the alpine meadow, indicating that the alpine meadow vegetation conformed to the pattern of allometric growth (Figure 2 and Table 2). On the QTP, the aboveground and belowground parts of the alpine meadow vegetation showed different plant growth patterns, in that, the aboveground parts showed isometric growth, while the belowground parts showed allometric growth (Xu and Xue, 2013). After warming, the aboveground and belowground parts showed an allometric growth pattern indicating that warming enhanced the allometric growth pattern in the alpine meadow vegetation (Table 2).

Studies on different growth forms of plants found that the aboveground and belowground biomass allocation of arbor and grass plants were all consistent with the pattern of isometric growth, such as evergreen broadleaved forest (Fei et al., 2016), secondary mixed forest (He et al., 2016), Chinese fir plantation (Fei et al., 2016), and *Loropetalum chinense* (Wang Y. et al., 2017), while the shrub plants showed the pattern of allometric growth, such as *Horaninowia ulicina* and *Alhagi sparsifolia* (Zhang et al., 2019). These results indicated that the plant growth patterns were different among different plant species. For different individuals of *Loropetalum chinense*, the growth pattern between vegetative organs changed from isometric to allometric growth with an increase in the number of individuals in a plot (Wang Y. et al., 2017). Therefore, the type of growth pattern also changed with variations in plant density.

In addition, the transformation of the plant growth patterns was also related to the allocation of biomass, and the production and allocation of biomass were greatly affected by precipitation and temperature. Therefore, the transformation of the plant growth patterns was subject to the effects of climate warming (Xu and Xue, 2013; Liu, 2017). Studies have shown that decreased precipitation could increase the proportion of biomass allocated to the belowground parts (Liu et al., 2021). With an increase in temperature, the vegetation height and coverage of the alpine meadow vegetation increased (Zhao Y. Y. et al., 2015), and more biomass was allocated to the aboveground parts (Zhang and Peng, 2018), but studies from Ma L. et al. (2020) showed that the increase in the height was accompanied by a decrease on the aboveground biomass of the alpine meadow vegetation. Xu and Xue (2013) found that the aboveground parts of the alpine meadow vegetation conformed to the pattern of isometric growth, while the belowground parts exhibited allometric growth, and in general, the vegetation had an allometric growth pattern in the alpine meadow. We also found similar results in the control treatment in this study, i.e., the plant growth patterns were different for the aboveground and belowground parts of the alpine meadow vegetation due to large differences in the climate (Table 2). Furthermore, Liu (2017) found that an increase of moisture changed the growth pattern of *Salix phellodendron*; that is, the relationship between branch length and basal diameter presented the pattern of allometric growth in mobile sandy land, and then adjusted to isometric growth in semi-fixed and fixed sandy land, and lastly changed to allometric growth again in the inter-dune lowland. Therefore, the plant growth patterns could be transformed by the climate (especially the precipitation and temperature).

It could be proved that most of the growth patterns among the vegetation organs conformed to the pattern of allometric growth. However, owing to the differences in plant functional types, species composition, individual density, and climate conditions, the plant growth patterns were transformed between isometric and allometric growth patterns (Liu, 2017). Therefore, for the alpine meadow in this study, the transformation of the growth patterns on the aboveground parts from isometric to allometric growth under the treatment of warming was likely to be caused by the increase in temperature and by the weakened correlations of the plant growth indices with soil stoichiometric characteristics.

### Response of Soil Stoichiometric Characteristics to Warming

Warming enhanced the microbial activity and then accelerated the decomposition rate of the soil nutrients, so the soil nutrient content decreased (Zhou et al., 2013; Waqas et al., 2021). In this study, we found that the C, N, and P contents at the depth of 0–20 cm decreased under the warming treatment (Figure 3 and Table 3), indicating that warming promoted the decomposition of organic matter, resulting in a decrease on the C and N contents, which was consistent with the conclusions from Jiang et al. (2014) and Ouyang et al. (2018). Meanwhile, we also found that the C, N, and P contents at the depth of 20–30 cm increased under the warming treatment (Figure 3 and Table 3), because the increase of the plant root biomass tended to occur in the deeper soil layer with warming (Xu et al., 2016), and the decomposition of the deeper roots increased the contents of C, N, and P in the deep soil. Therefore, warming resulted in changes to the distribution of C, N, and P in soils at various depths and increased their concentrations at the deep layers.

The same trend also existed in the variations of soil C, N, and P stoichiometric ratios during warming (Figure 3 and Table 3). Soil C, N, and P stoichiometric ratios were an important link in the nutrient cycles between soil and plant ecosystems (Zhan et al., 2017), and also served as an important indicator of C, N, and P cycles among vegetation, litter, and soil (Chen and Chen, 2021). In studies from Qin et al. (2020), soil C:N, C:P, and N:P increased with a warming treatment, and the amplitudes of change increased with depth, indicating that warming promoted the C:N, C:P, and N:P to change so that the deep soil had higher levels of these nutrients than the shallow soil. Our study also found that the C:N at each soil depth was very stable under the warming treatment (Table 3), indicating that soil C and N were not sensitive to the warming effects (Ning et al., 2019) and could maintain a relatively stable ratio in the soil. Meanwhile, for P, the effects of warming on the C:P and N:P were greater at the 20–30 cm depth than at the 0–10 cm depth (Table 3), indicating that warming accelerated the loss of P in the soil. Therefore, the C:P and N:P ratios could be used as important indicators for the restriction of nutrients in the alpine meadow. Dong et al. (2019) also had a similar conclusion in a study of soil stoichiometry of shrubland. In turn, the downward migration of the soil stoichiometric characteristics, caused by warming, had effects on the growth of vegetation in the alpine meadow.

### Relationship Between Plant Growth and Soil Stoichiometric Characteristics in Warming

In the alpine meadow vegetation, an increase in the temperature promotes growth (Ma et al., 2017). In this study (Figure 4 and Table 4), the soil stoichiometric characteristics facilitated the growth of plants in an alpine meadow, but the correlations between the plant growth and soil stoichiometric characteristics were weakened by warming, as the downward migration of the soil stoichiometric characteristics in warming exerted influences on the growth of vegetation.

Warming enhanced the relationships of plant density with soil N content at the 10–30 cm depth, P content at the 20–30 cm depth, and N:P at the 10–20 cm depth (Table 4), explaining that warming promoted increases in the contents of N and P into deep soil, and thus provided nutrients to the deep soil, which increased the number of plants. This conclusion was proven by Wang (2019), in that, warming increased the species richness and density of alpine meadow seedlings by increasing the contents of soil N and P. Moreover, a positive correlation was also observed between plant belowground biomass and soil C content at the 20–30 cm depth (Table 4), indicating that warming caused an increase in the distribution of roots to the deep soil on the alpine meadow, which was consistent with the conclusion from Li et al. (2011) that the effects of warming resulted in a strengthening of root biomass in deep soil. Xu et al. (2015a) also concluded that the drought caused by the downward migration of soil moisture in warming altered the allocation of the plant belowground biomass at various soil depths; this allocation of plant belowground biomass to deeper layers modified the distribution of C and N in the soil of the alpine meadow. Therefore, for different soil depths, warming mainly enhanced the correlations of the plant growth with soil stoichiometric characteristics at the 10–30 cm depth, which further proved that the downward migration of the soil stoichiometric characteristics to deep soil in warming had influenced the growth of the aboveground vegetation in the alpine meadow.

Plant height and coverage shared a similar trend in the use of resources, such as light and nutrients. In this study, we found that a significant positive correlation existed between plant height and soil N and P contents in the deep soil (Table 4). Wang et al. (2014) found that warming increased grass height and biomass in the alpine grasslands. Zhao Y. Y. et al. (2015) also found that warming increased the height of the meadow plants, but the effects in coverage were not obvious. The increase in soil temperature accelerated the decomposition of organic matter by soil microorganisms and increased soil mineralization, thus improving the soil nutrient contents and promoting plant growth (Nadelhoffer et al., 1991). However, we found that warming weakened the correlations between plant height and coverage with the soil stoichiometric characteristics (Table 4). The reason for this difference might be related to the warming duration, the geographical location of the study area, and the type of plant species.

Soil C, N, and P contents and their stoichiometric ratios reflect the nutrient status and limitation patterns under increasing temperatures (He et al., 2008; Ning et al., 2019). In this study, we found that the correlations of plant growth with the soil stoichiometric characteristics were weakened by warming (Figure 4 and Table 4), demonstrating that the downward migration of the soil stoichiometric characteristics in warming created effects on the growth of vegetation in the alpine meadow. This conclusion was the same as others as follows. Yu et al. (2015), Liu (2018), and Jia et al. (2019) concluded that warming promoted the downward migration of soil stoichiometry and affected the pattern of allometric growth in deeply rooted plants. Yang et al. (2015) believed that with an increase in the warming time, the warming effects of warming weakened and resulted in the decrease of the soil C, N, and P contents. Zhao Y. Y. et al. (2015) found that warming accelerated the decomposition of organic matter resulting over time in reduced N and P contents in an alpine meadow soil. Domisch et al. (2002) and Li et al. (2010) believed that warming reduced the soil stoichiometric contents. The study of Heng et al. (2011) also showed that warming promoted soil nutrient cycling and decreased the soil C, N, and P contents. We conclude that a close relationship exists between the plant growth and soil stoichiometric characteristics in alpine meadows, which was weakened by the increase in temperature over time. The downward migration of the soil stoichiometric characteristics in warming promoted the allometric growth pattern in the vegetation of the alpine meadow.

## Conclusion

We report for the first time the results of the plant allometric growth and soil stoichiometric characteristics from experimental warming in an alpine meadow of the QTP. Warming enhanced the pattern of the plant allometric growth and promoted migration of the soil stoichiometric characteristics to deep layers. The downward migration of the soil stoichiometric characteristics, caused by warming, influenced the amount of allometric growth observed in the plants in the alpine meadow. Our study complements the earlier reports that climate warming could affect the distribution of biomass and nutrients in the soil. The downward migration of moisture, driven by increased radiation at the surface, was probably responsible for the distribution of plant belowground biomass and nutrients to the deeper soil levels. Therefore, we successfully verified the hypothesis that the plant allometric growth was influenced greatly by the downward migration of the soil stoichiometric characteristics to deep layers of the alpine meadow under climate warming. Although the uncertainties remain related to the artificial simulation of climate warming, global warming is likely to change the soil conditions and will affect plant growth in particular in permafrost environments.

## Data Availability Statement

The original contributions presented in the study are included in the article/supplementary material, further inquiries can be directed to the corresponding author/s.

## Author Contributions

MX and HZ performed the experiments. HZ and LM contributed technical advice in equipment installation. MX contributed to the collection of the data and writing the first draft. HZ, ZZ, LM, and XL contributed to the writing—reviewing, editing, and supervision. ZZ, LM, and XL provided assistance during data processing and data analysis. All authors contributed to the article and approved the submitted version.

## Conflict of Interest

The authors declare that the research was conducted in the absence of any commercial or financial relationships that could be construed as a potential conflict of interest.

## Publisher’s Note

All claims expressed in this article are solely those of the authors and do not necessarily represent those of their affiliated organizations, or those of the publisher, the editors and the reviewers. Any product that may be evaluated in this article, or claim that may be made by its manufacturer, is not guaranteed or endorsed by the publisher.
